# Physiological and Psychological Stress of Microwave Radiation-Induced Cardiac Injury in Rats

**DOI:** 10.3390/ijms24076237

**Published:** 2023-03-25

**Authors:** Dayan Li, Xinping Xu, Yue Yin, Binwei Yao, Ji Dong, Li Zhao, Haoyu Wang, Hui Wang, Jing Zhang, Ruiyun Peng

**Affiliations:** Beijing Institute of Radiation Medicine, Beijing 100850, China

**Keywords:** microwave radiation, the thermal effect, physiological and psychological stress, JNK pathway, cardiac

## Abstract

Electromagnetic waves are widely used in both military and civilian fields, which could cause long-term and high-power exposure to certain populations and may pose a health hazard. The aim of this study was to simulate the long-term and high-power working environment of workers using special electromagnetic radiation occupations to clarify the radiation-induced stress response and cardiac damage and thus gain insights into the mechanisms of injuries caused by electromagnetic radiation. In this study, the combination of microwave and stress was an innovative point, aiming to broaden the research direction with regard to the effect and mechanism of cardiac injury caused by radiation. The myocardial structure was observed by optical and transmission electron microscope, mitochondrial function was detected by flow cytometry, oxidative-stress markers were detected by microplate reader, serum stress hormone was detected by radioimmunoassay, and heart rate variability (HRV) was analyzed by multichannel-physiological recorder. The rats were weighed and subjected to an open field experiment. Western blot (WB) and immunofluorescence (IF) were used to detect the expressions and distributions of JNK (c-Jun N-terminal kinase), p-JNK (phosphorylated c-Jun N-terminal kinase), HSF1 (heat shock factor), and NFATc4 (nuclear factor of activated T-cell 4). This study found that radiation could lead to the disorganization, fragmentation, and dissolution of myocardial fibers, severe mitochondrial cavitation, mitochondrial dysfunction, oxidative-stress injury in myocardium, increase to stress hormone in serum, significant changes in HRV, and a slow gain in weight. The open field experiment indicated that the rats experienced anxiety and depression and had decreased exercise capacity after radiation. The expressions of JNK, p-JNK, HSF1, and NFATc4 in myocardial tissue were all increased. The above results suggested that 30 mW/cm^2^ of S-band microwave radiation for 35 min could cause both physiological and psychological stress damage in rats; the damage was related to the activation of the JNK pathway, which provided new ideas for research on protection from radiation.

## 1. Introduction

With the progress of science and technology, electromagnetic waves of various wavelengths and frequencies have been flooding our living and working spaces [1]. Electromagnetism is involved in long-term and high-power applications in communication, medical, transportation, and military fields, and people were unknowingly exposed to radiation environment for a long time, which caused health hazards. The aim of this study was to simulate the long-term and high-power working environment of people in special electromagnetic radiation occupations to clarify the resulting radiation-induced stress response and heart damage, thus broadening the research direction regarding the damage mechanism and providing new targets for research into protection from radiation. A number of studies have shown that excessive electromagnetic radiation could cause damages in multiple organs of an organism [2], mediated through both thermal and non-thermal effects. At present, research on the non-thermal effects has been relatively extensive, though research on the thermal effect has still been relatively scarce. The injury mechanisms of the non-thermal effects, such as energy metabolism disturbance and oxidative stress injury, have been largely clarified [3], while the thermal effect injury mechanisms have not been systematically studied. This study aimed to explore the injury mechanisms involved in the thermal injury effects of electromagnetic radiation.

As one of the most sensitive target organs of electromagnetic radiation [4], the heart is the organ of circulatory driving force with active and finely coordinated electrical activity. It is extremely sensitive to nervous and endocrine regulation, as shown by the fact that the electrical activity of the heart is directly correlated to the sympathetic-adrenal and parasympathetic activities, and certain hormones are important triggering factors of cardiac diseases. Neuroendocrine disorders can lead to not only cardiac injury, but also the physiological stress reaction, which suggests that cardiac injury and stress may be closely related. The relationship between stress and cardiac structural and functional changes after electromagnetic radiation has not been carefully studied. The stress response is a non-specific response of the body to a stimulus. When the thermal effect of electromagnetic radiation accumulates to a certain extent, the body is thermally stressed. The heat stress response is mainly caused by heat accumulation and resultant dehydration and electrolyte disturbance, etc. Stress response induced by electromagnetic radiation has not been systematically studied, other than through oxidative stress, i.e., changes in peroxides and antioxidant enzymes. Therefore, this study aimed to study the damage and mechanism of the thermal effects after radiation from the perspective of stress. This could be a breakthrough in the direction of research regarding electromagnetic radiation-induced cardiac injury.

There is currently a shortage of study regarding the biological effects of electromagnetic radiation on the mechanism of stress damage, and there is no relevant research on how to evaluate the severity of stress-induced cardiac injury. Therefore, it was proposed that we use electromagnetic waves to radiate rats and to classify indicators based on physiological and psychological stresses. The degree of physiological stress-induced injury was evaluated by indicators that would reflect the structures and functions of the heart [5], and body weight. An open field experiment was used as the gold standard for evaluating whether the psychological stress model was successfully established [6,7,8], so as to clarify the successful construction of the stress model at the physiological and psychological levels, and to lay a groundwork for the research on the mechanism of heat stress.

In mechanism studies, electromagnetic radiation can significantly increase the expression of heat shock protein 72 (Hsp72) in rats’ myocardium. Hsp72 can resist heat stress injury, thereby inhibiting the expression and activation of stress marker protein JNK. However, when the stress response reaches a certain extent of severity, the regulatory effect of Hsp72 will fail and the JNK pathway will be activated. It has been widely reported in the literature that the JNK pathway can be activated thermal stimulation [9,10]. HSF1 downstream of JNK also has a transactivating ability [11] and could be activated under stress caused by thermal stimulation; in addition, NFATc4 downstream of JNK is closely related to Ca^2+^ transport [12] and could regulate electrophysiological function of the heart. Therefore, JNK, p-JNK, HSF1 and NFATc4 were selected for this study to investigate the mechanism of stress injury, which could provide insights on the protection of electromagnetic radiation.

## 2. Results

### 2.1. Structural Damage to Myocardial Tissue

**Figure 1:**
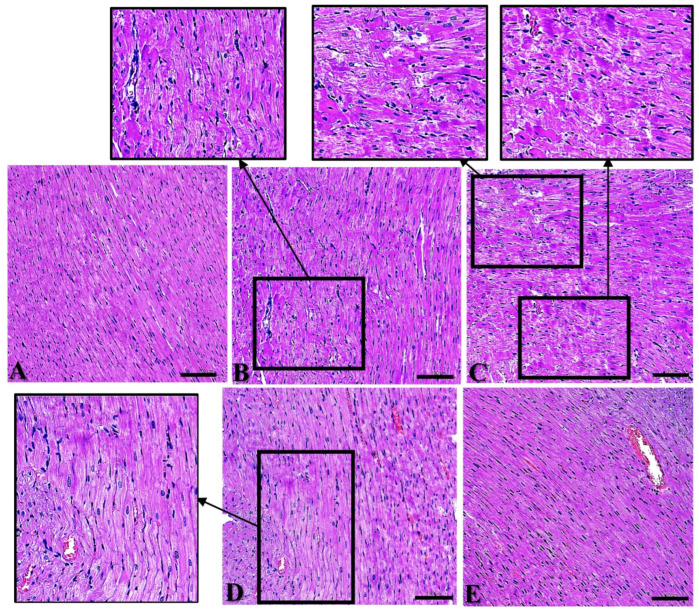
Structural changes to rat’s myocardium after S-band exposure. (**A**) Group C; (**B**) Group R on Day 1; (**C**) Group R on Day 7; (**D**) Group R on Day 14; (**E**) Group R on Day 28. (**A**–**E**) ×400, scale bar = 100 μm (black frames indicate wavy myocardial fibers and damaged areas).

In this study, Days 1, 7, 14, and 28 after exposure were set for dynamic observation of the myocardial structure, with the intent being to explore the law of temporal change after radiation. Within 28 d after exposure, cardiac muscle fibers in Group C were closely and neatly arranged, with rare myocardial fibrosis and rupture, and the nuclei were regular in shape with rare pyknosis and hyperchromia (Figure 1A). On Day 1 after radiation, myocardial fibers in Group R were disordered and wavy, with irregular nuclei, pyknosis, and hyperchromasia (Figure 1B); on Day 7 after radiation, cardiac muscle fibers were disorganized, dissolved, fractured, and loosely arranged (Figure 1C); on Day 14 after radiation, myocardial fibers were more closely arranged than on Days 1 and 7 after radiation, and there were still a few wavy myocardial fibers (Figure 1D); on Day 28 after radiation, myocardial fibers were arranged tidily and tightly, and the damage was basically restored (Figure 1E). The above results clarified that the most significant time points of structural damage in this model were on Days 1 and 7. A recovery trend appeared on Day 14 and damage had largely recovered by Day 28.

### 2.2. Ultrastructural Abnormalities in Myocardial Tissue

**Figure 2:**
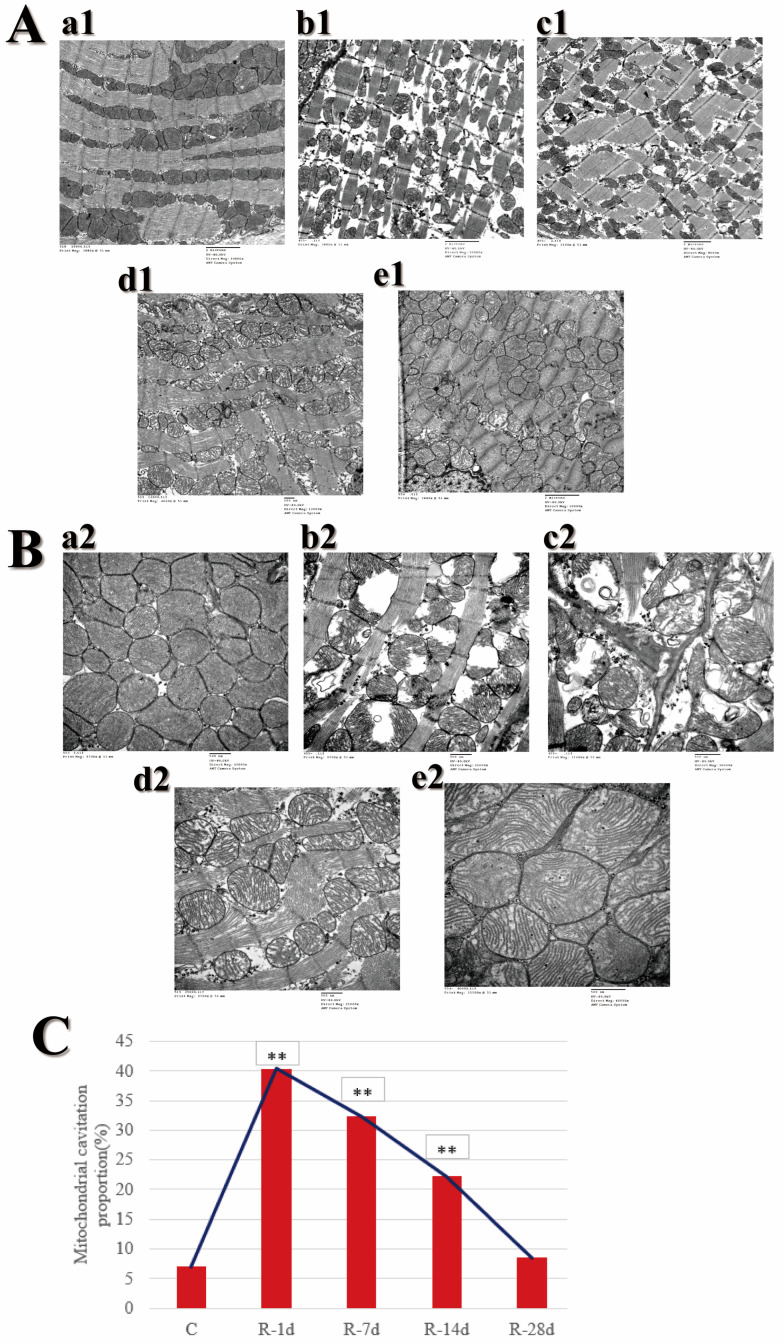
Ultrastructural changes of rats’ myocardium after S-band radiation. (**A**) Myocardial fibers: (**a1**) Group C (×10,000), (**b1**) Group R on Day 1 (×10,000), (**c1**) Group R on Day 7 (×8000), (**d1**) Group R on Day 14 (×12,000), (**e1**) Group R on Day 28 (×10,000). (**B**) Mitochondria: (**a2**) Group C (×25,000), (**b2**) Group R on Day 1 (×25,000), (**c2**) Group R on Day 7 (×30,000), (**d2**) Group R on Day 14 (×25,000), (**e2**) Group R on Day 28 (×40,000). (**C**) Statistical plot of the proportion of mitochondrial cavitation. Significance signs were classified according to *p*-value: ** *p* < 0.01.

The heart is the energy supply organ of the circulating system, pumping blood for the whole body through systole and diastole; it has a great energy demand, so it is rich in myofibrils and mitochondria. According to the previous study conducted by our group, this experiment chose Days 1, 7, 14, and 28 after radiation for dynamic observation of ultrastructure in myocardium, expecting to determine the time-changing laws of mitochondrial and myocardial fiber damage in rat’s myocardium after radiation. Figure 2A,B shows the changes in myocardial fibers and mitochondria.

Within 28 d after radiation, the cardiac muscle fibers in Group C were arranged orderly and the Z-line was intact under TEM (Figure 2(Aa1)). In Group R, the cardiac muscle fibers were loosely arranged and dissolved or even broken, and the Z-line was fractured on Day 1 after radiation (Figure 2(Ab1)); the injury had a recovery trend, but the cardiac muscle fibers were still dissolved and the Z-line was distorted and blurred on Day 7 after radiation (Figure 2(Ac1)); on Day 14 after radiation, the damage recovered significantly, the myocardial fibers were basically intact though still slightly dissolved, and the Z-line was basically intact though with partial blurring (Figure 2(Ad1)); myocardial fiber damage had basically recovered by Day 28 after radiation (Figure 2(Ae1)).

Within 28 d after radiation, the mitochondria of Group C were more numerous and closely arranged and structurally intact under TEM (Figure 2(Ba2)). In Group R, the number of mitochondria decreased, their shape and size varied, and the mitochondria had suffered severe swelling and cavitated on Day 1 after exposure(Figure 2(Bb2)); the mitochondria were severely damaged with no recovery trend on Day 7 after radiation (Figure 2(Bc2)); the mitochondrial damage recovered significantly, but the cristae were still broken on Day 14 after radiation (Figure 2(Bd2)); the mitochondria were more numerous and closely arranged, and the damage was basically completely recovered on Day 28 after radiation (Figure 2(Be2)). The results of statistical analysis in Figure 2C showed that radiation could cause an increase in the proportion of mitochondrial cavitation, which was markedly different from Group C on Days 1, 7, and 14 after radiation. The damage was the most severe on Day 1, and the damage was basically recovered only by Day 28.

Through ultrastructural observation, we found that the mitochondrial structure in the myocardium of rats after radiation was severely damaged and slow to recover, which provided an idea for further research. The damage to mitochondria in the myocardium can lead to energy metabolism disorder, and insufficient energy supply affects the systolic and diastolic function of the heart, thus causing disturbance of cardiac electrical activity. Therefore, mitochondrial function and cardiac electrophysiological function were the focus of future research.

### 2.3. Myocardial Mitochondrial Dysfunction of Rats

**Figure 3:**
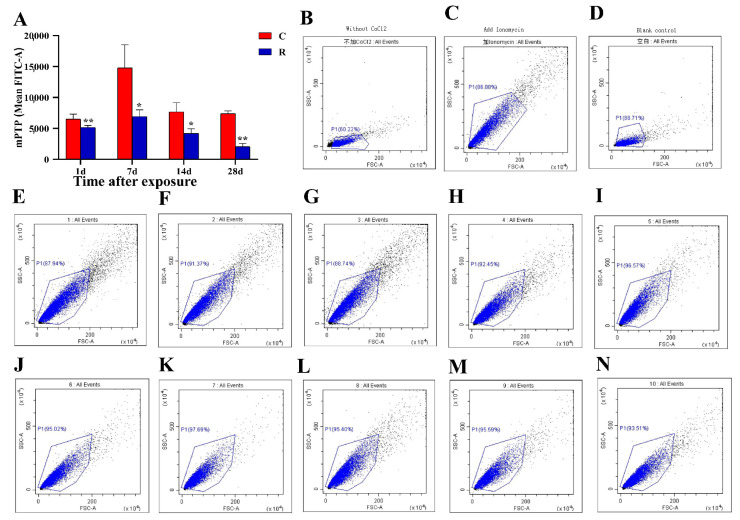
Changes in myocardial mPTP in rats after S-band radiation. (**A**) Statistical analysis graph; (**B**) flow diagram of negative control on Day 14; (**C**) flow diagram of positive control on Day 14; (**D**) flow diagram of blank control on Day 14; (**E**–**I**) flow diagrams of Group C on Day 14; (**J**–**N**) flow charts of Group R on Day 14. Significance signs were classified according to *p*-value: * *p* < 0.05 and ** *p* < 0.01.

The results of electron microscopy showed that the myocardial mitochondrial structure was significantly damaged in this model, and the structure and function corresponded, so the mitochondrial function was evaluated in this study. The mean fluorescence of mPTP in Group R was significantly decreased on Days 1, 7, and 14 after radiation (*p* < 0.05 or 0.01), and there was no remarkable change on Day 28 after radiation (Figure 3A) (*p* > 0.05); this indicated that the mPTP was damaged and still not fully recovered on Day 14. Additionally, the damage to mPTP led to the uncoupling of the electron transport chain and a decrease in MMP.

**Figure 4:**
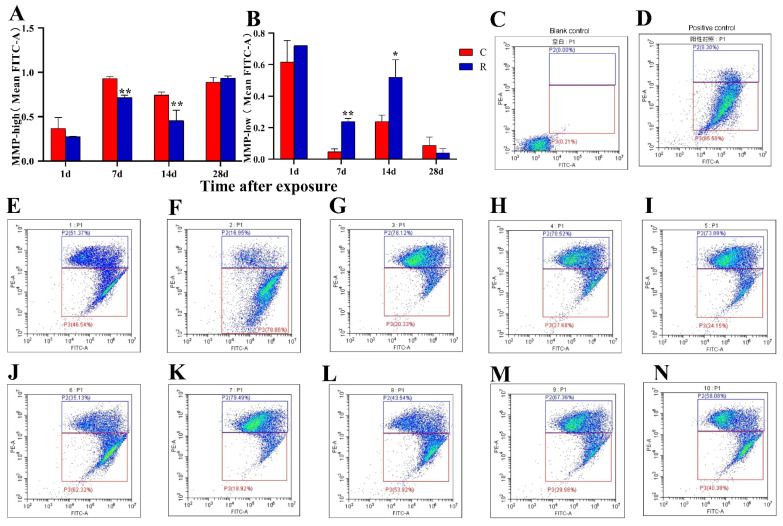
Changes in myocardial MMP in rats after S-band radiation. (**A**) Statistical analysis plot of MMP-high; (**B**) statistical analysis plot of MMP-low; (**C**) flow graph of blank control on Day 14; (**D**) flow graph of positive control on Day 14; (**E**–**I**) flow graphs of Group C on Day 14; (**J**–**N**) flow graphs of Group R on Day 14. Significance signs were classified according to *p*-value: * *p* < 0.05 and ** *p* < 0.01.

MMP-high values represent the proportion of live cells, and MMP-low values represent the proportion of damaged cells. MMP could also reflect the ability of mitochondria to synthesize ATP. This study found that the MMP-high (Figure 4A) in the Group R remarkably decreased on Days 7 and 14 after radiation (*p* < 0.01), while MMP-low (Figure 4B) significantly increased on Days 7 and 14 after radiation (*p* < 0.05); there were no marked changes at the other time points (*p* > 0.05). The experimental results for mPTP and MMP showed mitochondrial dysfunction in the myocardium of this model and suggested that the possible existence of impaired energy metabolism and electrophysiological impairment, but more in-depth experimental verification was needed.

### 2.4. Elevated Levels of Oxidative Stress in Rats’ Myocardium

**Figure 5:**
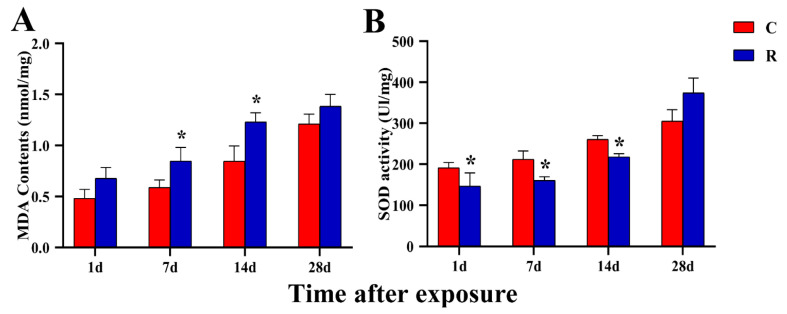
Changes in levels of rats’ myocardial oxidative stress after S-band radiation. (**A**) MDA content; (**B**) SOD activity. Significance signs were classified according to *p*-value: * *p* < 0.05.

Mitochondria play a major role in ROS generation, and mitochondrial damage will inevitably lead to the accumulation of ROS. Therefore, this study detected the levels of oxidative stress-related markers in myocardial tissue, and found that the MDA content of the Group R (Figure 5A) was remarkably higher than that of the Group C on Days 7 and 14 after radiation *(p* < 0.05), while there were no significant changes at other time points (*p* > 0.05). The SOD activity of Group R (Figure 5B) was significantly decreased on Days 1, 7, and 14 after exposure (*p* < 0.05), and there was no significant difference on Day 28 (*p* > 0.05). MDA content and SOD activity are the most commonly used indicators to determine the level of oxidative stress. The simultaneous occurrence of elevated MDA content and reduced SOD activity suggested that oxidative stress damage to the myocardium caused by microwave radiation had occurred, and this was consistent with the laws of temporal changes in structural damage to myocardial tissue.

### 2.5. Overall Increase in Serum Stress Hormone Levels of Rats

**Figure 6:**
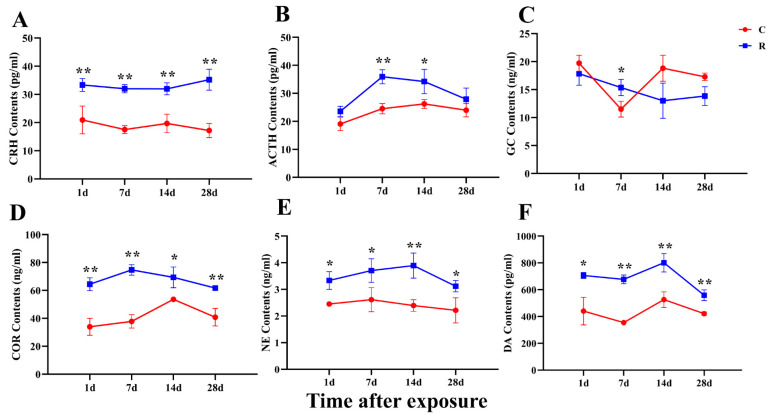
Changes in stress-related hormones in rats’ serum after S-band radiation. (**A**) CRH; (**B**) COR; (**C**) NE; (**D**) DA; (**E**) ACTH; (**F**) GC. Significance signs were classified according to *p*-value: * p < 0.05 and ** *p* < 0.01.

Changes in serum hormone levels are important evidence of stress and could reflect abnormal cardiac function. Therefore, this study detected the hormone levels in serum on Days 1, 7, 14, and 28 after radiation and found that the concentrations of CRH (Figure 6A), COR (Figure 6D), NE (Figure 6E), and DA (Figure 6F) in serum were significantly increased in the R group on Day 28 after radiation (*p* < 0.05 or 0.01). Additionally, ACTH (Figure 6B) increased significantly on Days 7 and 14 after radiation (*p* < 0.05 or 0.01), while GC (Figure 6C) was markedly increased on Day 7 after radiation (*p* < 0.05) and decreased on Day 28 after exposure (*p* < 0.05); there were no significant differences at other time points (*p* > 0.05). The above results indicated that radiation can cause a significant increase in hormone levels in serum, and hormones are an important material foundation for the occurrence of stress and can have an important impact on cardiac electrophysiological function. It was suggested that the stress caused by microwave radiation in rats was closely related to the changes in cardiac structure and function.

### 2.6. Increased Fluctuation in HRV in Rats

**Figure 7:**
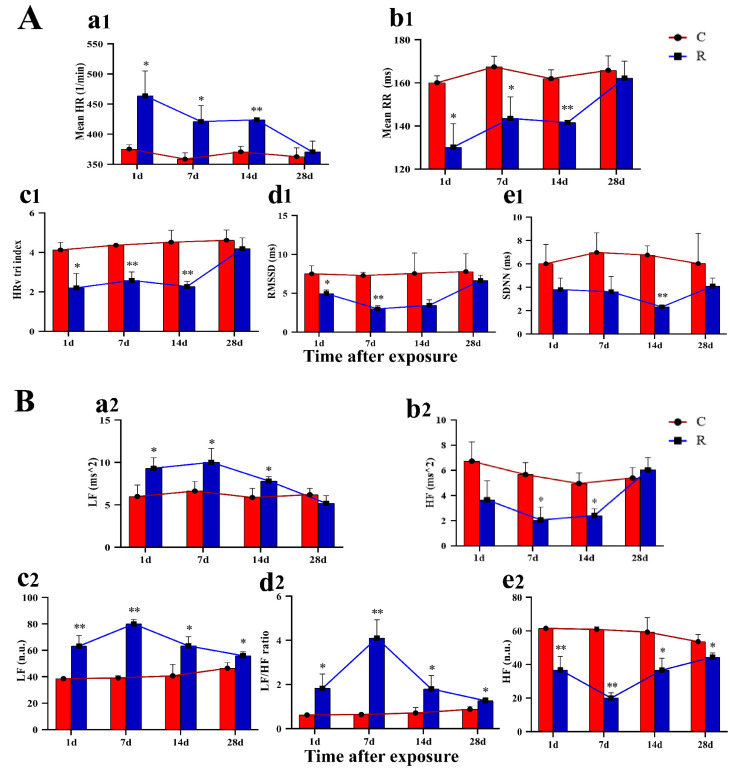
Changes in indicators related to HRV in rats after S-band radiation. (**A**) Time-domain related indicators: (**a1**) mean HR, (**b1**) mean RR, (**c1**) HRV tri-index, (**d1**) RMSSD, (**e1**) SDNN; (**B**) frequency-domain related indicators: (**a2**) LF-ms^2^, (**b2**) HF-ms^2^, (**c2**) LF-nu, (**d2**) LF/HF radio, (**e2**) LF-nu. Significance signs were classified according to *p*-value: * p < 0.05 and ** *p* < 0.01.

It has been shown that stress can lead to arrhythmias and even sudden cardiac death in severe cases [13]. However, as no studies have been conducted in the electromagnetic field, ECG were examined on Days 1, 7, 14, and 28 after radiation in this study, and relevant HRV indices were calculated by frequency-domain and time-domain analysis. The results of time-domain analysis of HRV were shown in Figure 7A. In Group R, mean HR (Figure 7(Aa1)) was significantly increased on Days 1, 7, and 14 after radiation, while mean RR (Figure 7(Ab1)) and HRV tri-index (Figure 7(Ac1)) decreased on Days 1, 7, and 14 after radiation (*p* < 0.05 or 0.01). RMSSD (Figure 7(Ad1)) decreased remarkably on Days 1 and 7 after radiation and SDNN (Figure 7(Ae1)) significantly decreased on Day 14 after radiation (*p* < 0.05 or 0.01). There was no significant differences at the rest of the time points (*p* > 0.05).

The results of the frequency-domain analysis of HRV are shown in Figure 7B. Compared with Group C, LF-ms^2^ in Group R (Figure 7(Ba2)) was significantly increased on Days 1, 7, and 14 after radiation *(p* < 0.05), and HF-ms^2^ (Figure 7(Bb2)) on Days 7 and 14 after radiation significantly decreased (*p* < 0.05), with no marked difference at the remaining time points (*p* > 0.05). LF-nu (Figure 7(Bc2)) and the LF/HF radio (Figure 7(Bd2)) were significantly increased, and HF-nu (Figure 7(Be2)) significantly decreased at four time points after exposure (*p* < 0.05 or 0.01). It has been established in the literature that HRV reflects the volatility of the autonomic nervous system [14] (ANS), and heart rate reflects the degree of tension in the ANS. The higher the individual stress level was, the greater the HRV volatility and the faster the heart rate changed. The results of this study showed that the heart rate of rats increased significantly, and the HRV fluctuated greatly, suggesting that the rats were in a state of stress due to the tension in the autonomic nervous system.

### 2.7. Significant Reduction in Body Weight of Rats

**Figure 8:**
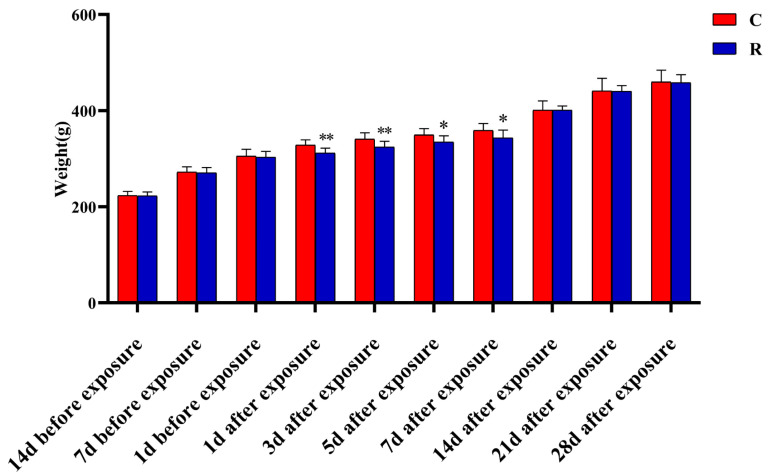
Weight changes in rats before and after S-band radiation. Significance signs were classified according to *p*-value: * p < 0.05 and ** *p* < 0.01.

The increase in serum stress hormone levels and the fluctuation in HRV in rats caused by microwave radiation might indicate that the rats in this model were psychologically stressed. Additionally, body weight can be used as a test to verify whether the psychological stress model is successfully established. This study found that the body weights of Group R (Figure 8) were not remarkably different from those of Group C before radiation (*p* > 0.05) but were remarkably lower than those of Group C on Days 1, 3, 5, and 7 after radiation (*p* < 0.05 or 0.01). There was no marked difference on Days 14, 21, and 28 after exposure (*p* > 0.05). The above results suggested that radiation can cause slow weight gain in rats, which verified that the psychological stress model had been successfully established to a certain extent.

### 2.8. Changes from the Open Field Experiment and Modification Behavior

**Figure 9:**
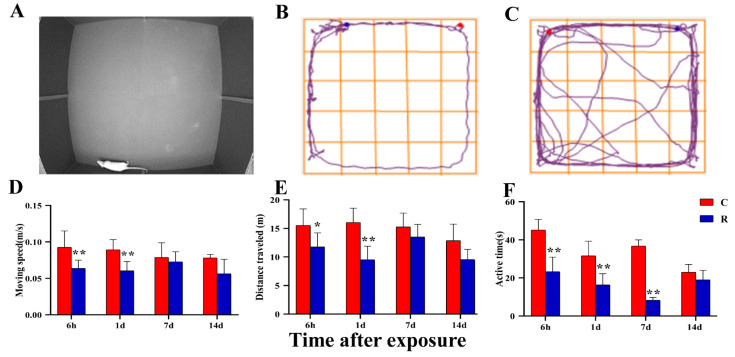
Changes noted during the open field experiment applied to rats after S-band radiation indicators of exercise ability. (**A**) The real shot of rats in the non-central area; (**B**) the trajectory diagram of Group R; (**C**) the trajectory diagram of Group C; (**D**) statistical analysis graph of moving speed; (**E**) statistical analysis graph of moving distance; (**F**) statistical analysis graph of active time. Significance signs were classified according to *p*-value: * p < 0.05 and ** *p* < 0.01.

In addition to body weight, the open field experiment is the gold standard for verifying the successful establishment of a psychological stress model. According to the time-varying laws of body weight, four time points of Hour 6, Day 1, Day 7, and Day 14 after radiation were established in the open field experiment. The real shot of rats in the non-central area is shown in Figure 9A, the movement trajectories of the rats in the Group R are shown in Figure 9B, and the movement trajectories of the rats in the Group C are shown in Figure 9C. Compared with Group C, the moving speed (Figure 9D) and moving distance (Figure 9E) of Group R were significantly decreased at Hour 6 and on Day 1 after radiation (*p* < 0.05 or 0.01), and the active time (Figure 9F) was significantly decreased at Hour 6, on Day 1, and on Day 7 after radiation (*p* < 0.01), but there was no noteworthy difference at other time points (*p* > 0.05). Moving speed, moving distance, and active time can all reflect the exercise ability of rats. The above results suggested that radiation can lead to a decline in the exercise ability and activity of rats.

**Figure 10:**
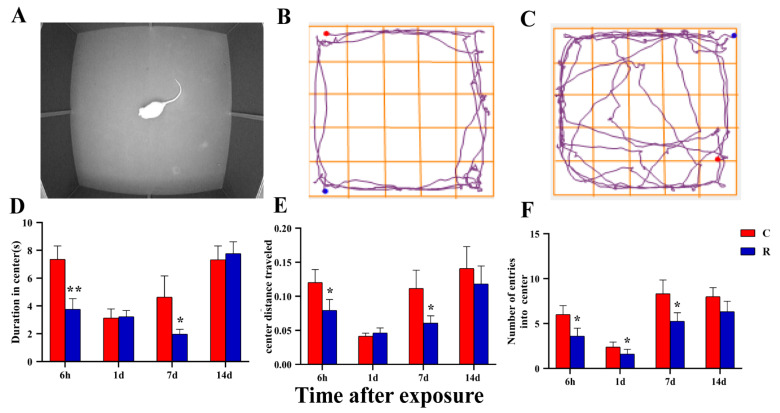
Changes noted during the open field experiment applied to rats after S-band radiation—indicators of exploring behavior. (**A**) The real shot of rats in the non-central area; (**B**) the trajectory diagram of Group R; (**C**) the trajectory diagram of Group C; (**D**) statistical analysis diagram of duration in the central region; (**E**) statistical analysis diagram of the ratio of the moving distance in the central region to the total distance; (**F**) statistical analysis graph of the number of times rats entered the central region. Significance signs were classified according to *p*-value: * p < 0.05 and ** *p* < 0.01.

The real shot of rats in the central area is shown in Figure 10A, the movement trajectories of rats in Group R is shown in Figure 10B, and the movement trajectories of rats in Group C is shown in Figure 10C. Compared with Group C, the dwell time in the central region (Figure 10D) and the central region movement-distance-to-total-distance ratio (Figure 10E) were significantly decreased in Group R at Hour 6 and on Day 7 after radiation (*p* < 0.05 or 0.01). The number of times rats entered the central area (Figure 10F) decreased significantly at Hour 6, on Day 1, and on Day 7 after exposure (*p* < 0.05), There were no significant differences at other time points (*p* > 0.05). The animals were so fearful and anxious in the new open environment that they mainly moved in the peripheral area. Therefore, the indicators related to the central area could reflect the anxiety of rats, and the above results suggest that radiation could cause anxiety in rats.

**Figure 11:**
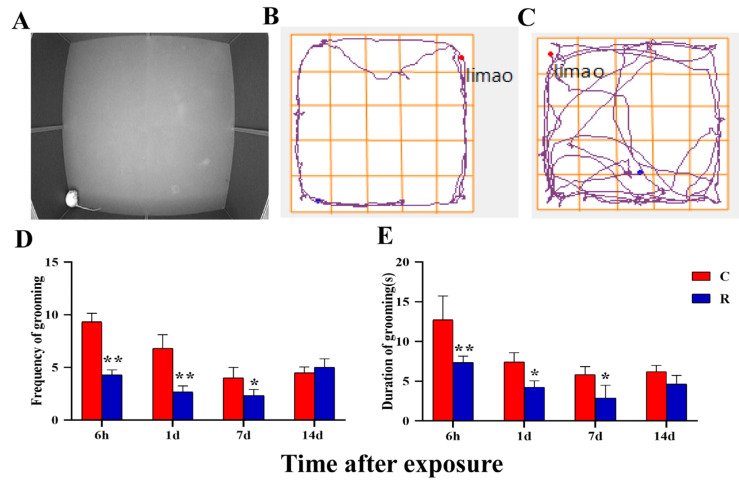
Changes in the open field experiment of rats after S-band radiation—grooming behavior. (**A**) The real shot of the rat when grooming; (**B**) the trajectory diagram of the Group R; (**C**) the trajectory diagram of the Group C; (**D**) statistical analysis graph of grooming frequency; (**E**) statistical analysis graph of grooming duration. Significance signs were classified according to *p*-value: * *p* < 0.05 and ** *p* < 0.01.

The real shot of rats’ grooming behavior is shown in Figure 11A, the movement trajectories of Group R are shown in Figure 11B, and those of Group C are shown in Figure 11C. Compared with Group C, the frequency and duration of grooming (Figure 11D,E) significantly decreased in Group R at Hour 6, on Day 1 and on Day 7 after radiation (*p* < 0.05 or 0.01), though there was no noteworthy difference at Day 14 after radiation (*p* > 0.05). Animals that are less anxious are more inclined to perform many relaxing actions, such as grooming and exploring unknown spaces, so grooming is an important modifying behavior that reflects anxiety and arousal in rats. The results regarding grooming frequency and duration suggested that radiation could lead to increased anxiety and decreased concentration in rats, which was consistent with the trends of other indicators in the open field experiment.

**Figure 12:**
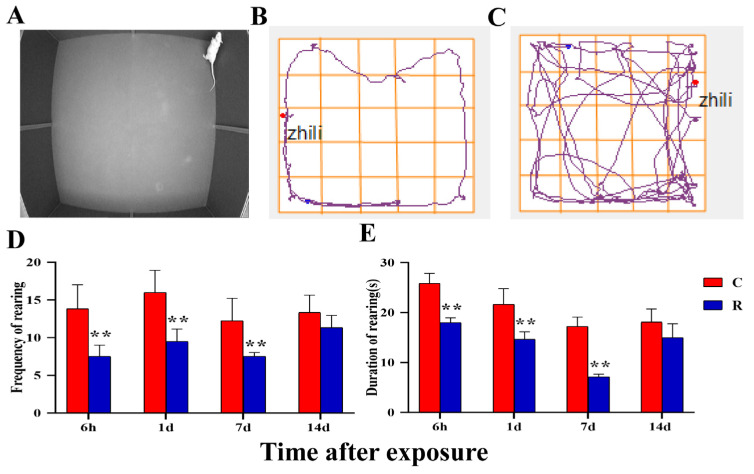
Changes noted during the open field experiment of rats after S-band radiation-upright behavior. (**A**) The real shot of the rat when upright; (**B**) the trajectory diagram of the Group R; (**C**) the trajectory diagram of the Group C; (**D**) statistical analysis graph of frequency of upright behavior; (**E**) statistical analysis graph of duration of upright behavior. Significance signs were classified according to *p*-value: ** *p* < 0.01.

The real shot of rats’ upright behavior is shown in Figure 12A, the movement trajectories of Group R is shown in Figure 12B, and those of Group C are shown in Figure 12C. Compared with Group C, the frequency and duration of upright behavior (Figure 12D,E) were significantly decreased in Group R at Hour 6, on Day 1, and on Day 7 after exposure (*p* < 0.05 or 0.01), though there was no marked difference on Day 14 after exposure (*p* > 0.05). The frequency and duration of upright behavior reflected the desire to explore the new environment and the anxiety and depression of rats. The results of this experiment showed that radiation could lead to a decrease in rats’ desire to explore and cause the emergence of anxiety and depression in rats.

### 2.9. Increased Expression of Myocardial Stress Proteins in Rats 

**Figure 13:**
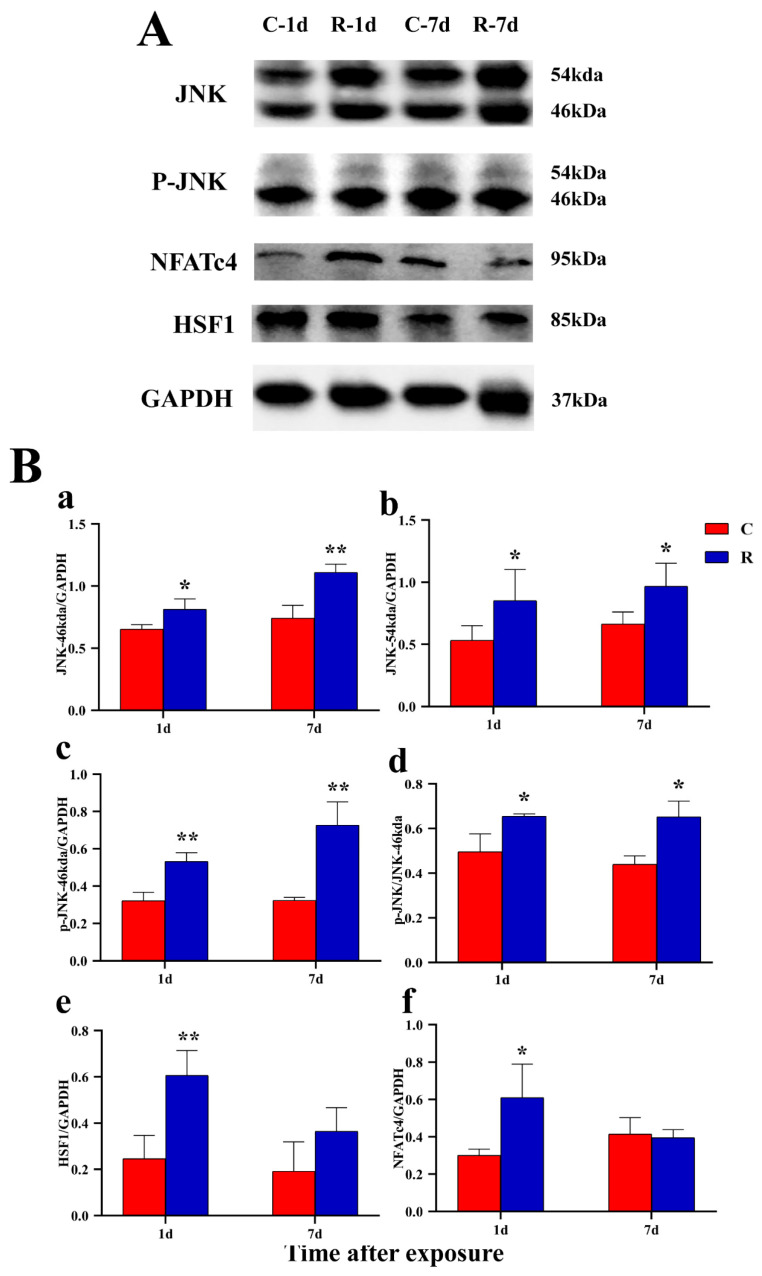
Changes in expression in rats’ myocardium after S-band radiation. (**A**) The original graphs of strips; (**B**) the graphs of statistical analysis:(**a**) JNK-46 kDa; (**b**) JNK-54 kDa; (**c**) p-JNK-46 kDa; (**d**) p-JNK/JNK; (**e**) HSF1; (**f**) NFATc4. Significance signs were classified according to *p*-value: * *p* < 0.05 and ** *p* < 0.01.

**Figure 14:**
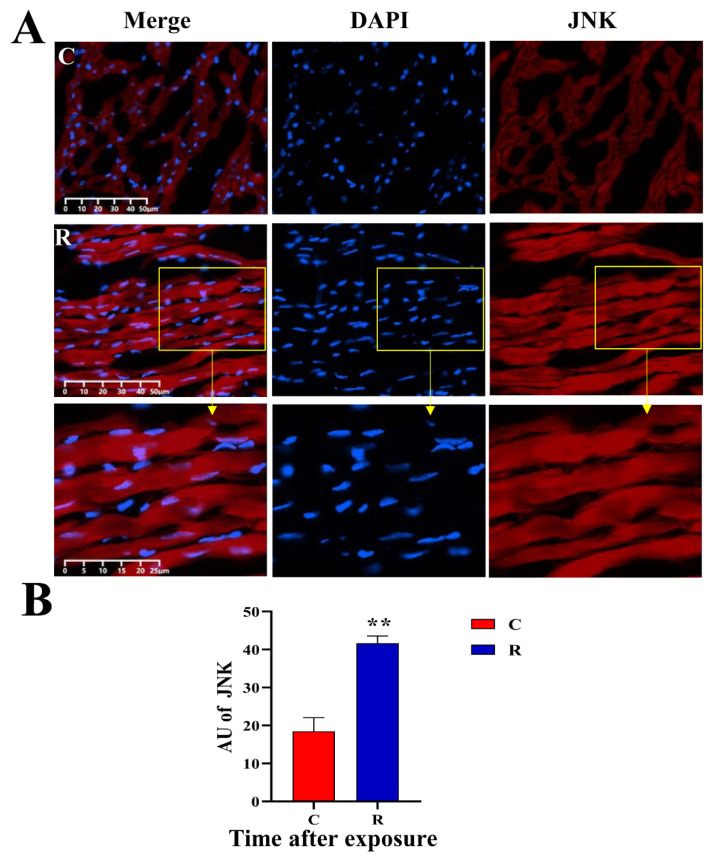
Changes in JNK expression and distribution of rats’ myocardium after S-band radiation. (**A**) Original graph of IF; (**B**) statistical analysis graph of average fluorescence intensity (AU). Significance signs were classified according to *p*-value: ** *p* < 0.01. The yellow box is the magnified area.

**Figure 15:**
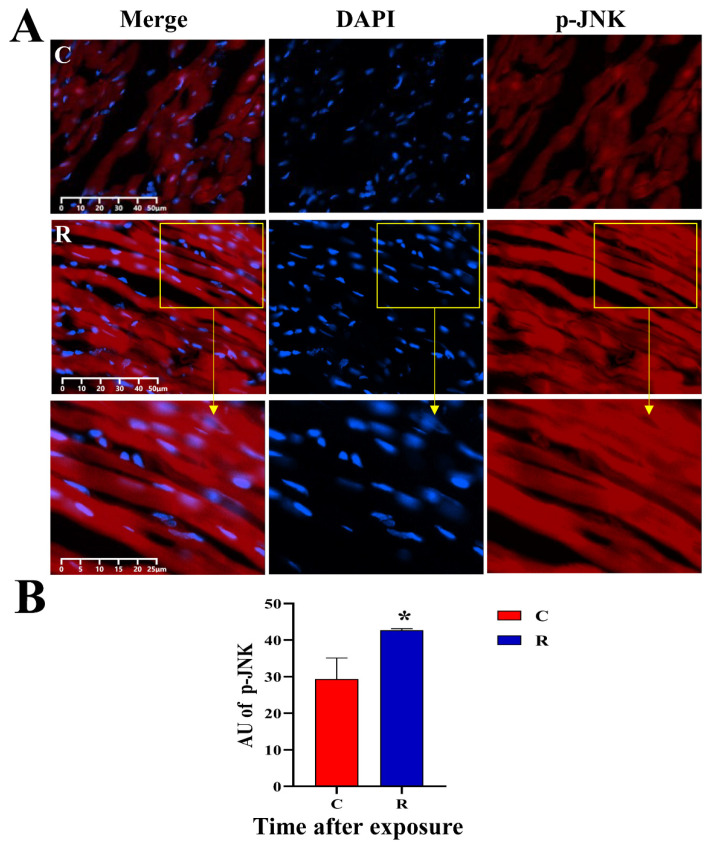
Changes in p-JNK expression and distribution of rats’ myocardium after S-band radiation. (**A**) Original graph of IF; (**B**) statistical analysis graph of AU. Significance signs were classified according to *p*-value: * p < 0.05. The yellow box is the magnified area.

**Figure 16:**
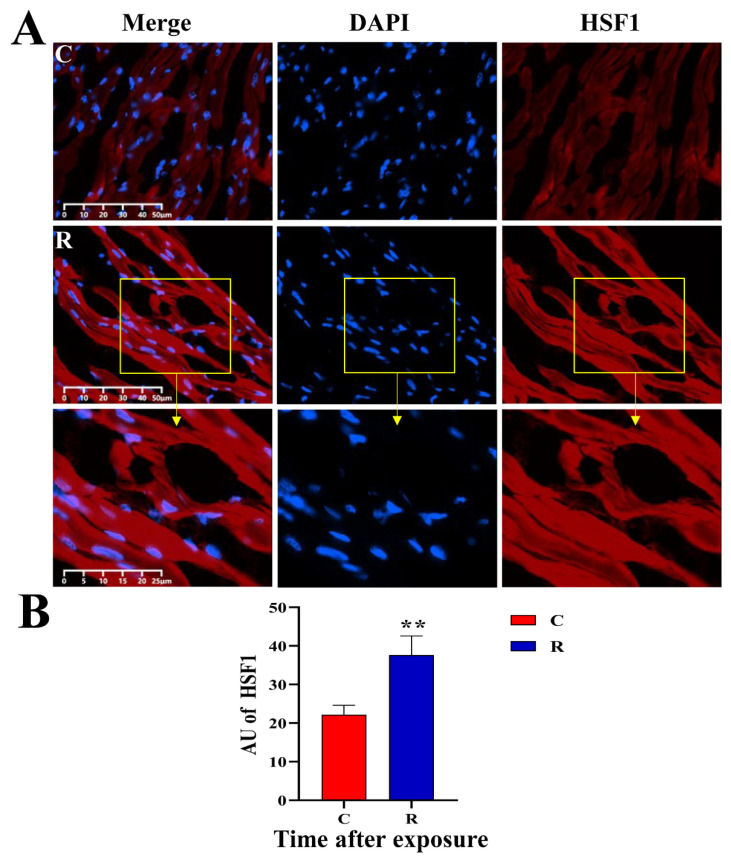
Changes in HSF1 expression and distribution of rats’ myocardium after S-band radiation. (**A**) Original graph of IF; (**B**) statistical analysis graph of AU. Significance signs were classified according to *p*-value: ** *p* < 0.01. The yellow box is the magnified area.

**Figure 17:**
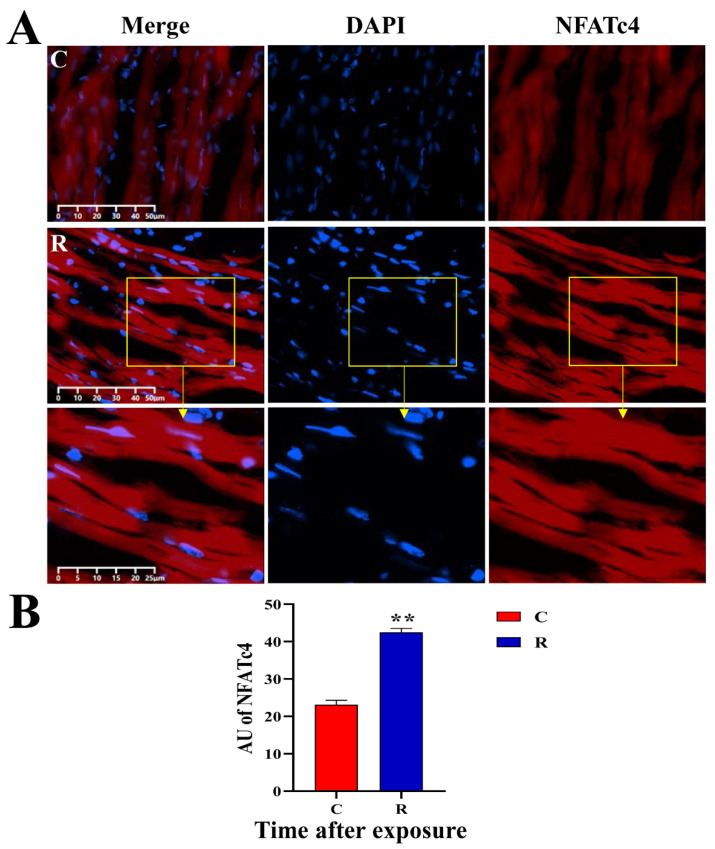
Changes in NFATc4 expression and distribution of rats’ myocardium after S-band radiation. (**A**) Original graph of IF; (**B**) statistical analysis graph of AU. Significance signs were classified according to *p*-value: ** *p* < 0.01. The yellow box is the magnified area.

The original images of WB strips are shown in Figure 13A. Compared with Group C, the JNK-46kDa (Figure 13(Ba)), JNK-54kDa (Figure 13(Bb)), p-JNK-46kDa (Figure 13(Bc)), and p-JNK/JNK-46kDa (Figure 13(Bd)) ratios in the myocardial tissue of rats in Group R were significantly increased on Days 1 and 7 after exposure (*p* < 0.05 or 0.01), which suggested that the activation of JNK was increased after radiation. The levels of HSF1 (Figure 13(Be)) and NFATc4 (Figure 13(Bf)) in Group R were significantly increased on Day 1 after exposure (*p* < 0.05 or 0.01), but showed no prominent change on Day 7 after radiation (*p* > 0.05). The results of IF showed that the red fluorescence in Figure 14, Figure 15, Figure 16 and Figure 17 was the labeled target protein, and JNK (Figure 14), p-JNK (Figure 15), HSF1 (Figure 16), and NFATc4 (Figure 17) were diffusely distributed in the nucleus and cytoplasm. The expression was markedly increased on Day 1 after exposure (*p* < 0.05 or 0.01), which was consistent with the results of WB.

The significant increase in the expression of HSF1 was consistent with the increase in the expression of Hsp72 in the previous study conducted by our group [15], indicating that the heat stress model was successfully established. Meanwhile, the significant increase in the expression of NFATc4 indicated the activation of the JNK pathway, suggesting the possible existence of disordered Ca^2+^-transport and impaired electrical cardiac conduction in this study [16].

## 3. Discussion

Electromagnetic waves are almost ubiquitous in modern life, of which S-band radiation is the electromagnetic wave band with a frequency of 2–4 GHz and is widely used in both civilian and military fields. Many advanced communication networks such as 5G and wireless routing use S-band microwaves, which expose people to microwave radiation for a long time without them knowing it, causing harm to health [2]. The heart is an organ that is sensitive to electromagnetic radiation [4] and is regulated by both neurological and endocrine systems. Since neuroendocrine disruption forms the physiological basis of stress, we hypothesized that stress could also lead to cardiac injury. However, there was no existing related research on microwave radiation-induced cardiac injury and stress. Therefore, this study took the combination of microwave and stress as an innovative point to broaden the direction for the study of the effect and mechanism of electromagnetic radiation-induced cardiac injury.

In this study, stress injury was divided into two categories: physiological stress and psychological stress. The indicators of physiological stress mainly reflected structural and functional damage to the heart, while the indicators of psychological stress reflected changes in mood and behavior. The degree of physiological stress injury was first assessed by optical and electron microscope to observe the structural damage in the myocardium, and then the mitochondrial function and the levels of oxidative stress, stress-related hormones, and HRV were detected to evaluate the cardiac functions of energy metabolism, anti-stress functions, and electrical conduction. Finally, this study used body weight and the open field experiment to evaluate whether the psychological stress model was successfully established. This study took the heart as the breakthrough point, comprehensively estimated the damage characteristics of stress reaction from the perspectives of physiology and psychology, and explored the association between heart injury and stress reaction caused by electromagnetic radiation.

Studies have shown that the heart’s contraction and blood flow require a lot of energy, so myocardial tissue is rich in myocardial fibers and mitochondria. A flood of studies have shown that microwave radiation can cause myocardial structural damage, especially mitochondrial structural damage [17,18]. It has also been reported that microwaves can lead to mitochondrial dysfunction. Picard M et al. [19] found that the occurrence of stress led to damage to mitochondrial structure, mPTP, and MMP [20]. The myocardial structure of this model was significantly damaged on Day 1 after radiation, and the damage was aggravated on Day 7 after exposure. It showed a recovery trend on Day 14 after exposure and was basically recovered on Day 28 after exposure. The main manifestations were: myocardial fibers were disordered in arrangement under optical microscopy; myocardial fibers were broken and dissolved, the Z-line was blurry; and mitochondria suffered swelling and were cavitated under electron microscopy. Corresponding to the mitochondrial structural damage, mitochondrial dysfunction was also found in this study, as evidenced by the decreased permeability of mPTP and abnormal MMP, suggesting an energy metabolism dysfunction in the heart, which was also one of the manifestations of physiological stress damage in this model.

Mitochondria, as energy factories, are also the main production sites of ROS, and the accumulation of ROS could lead to oxidative stress injury [21]. As a kind of physiological stress response, oxidative stress has been the most widely studied mechanism of electromagnetic injury. Kuchukashvili Z et al. [22] found that under psychological stress conditions, the antioxidant system was disturbed in the cardiomyocytes and blood of experimental rats, which manifested as the enhancement of the lipid peroxidation process and a decrease in the activity of antioxidative enzymes. This study also found that the content of MDA increased and the activity of SOD decreased after radiation, while the damage had recovered for the most part by Day 28 after radiation; this recovery time was basically the same as that of myocardial structure. We suggest that the existence of oxidative stress injury and the impairment of anti-stress function in myocardium tentatively verified the occurrence of physiological stress injury in this model.

Studies have shown that stress can cause neuroendocrine disorders, such as hyperactivity of the HPA axis and autonomic nervous dysfunction [23]. Most of the physiological and biochemical changes caused by the stress reaction are related to the excitation of the HPA axis, the sympathetic-adrenal medulla and parasympathetic nerve. After the HPA axis is activated, it can regulate the secretion of ACTH from the pituitary, the secretion of COR from the adrenal medulla, and the secretion of CRH from the paraventricular nucleus. The main function of CRH is to stimulate the secretion of ACTH from the pituitary after being accepted by the CRH receptor in the pituitary, and ACTH promotes the secretion of GC from the adrenal cortex [24]. GC can regulate the metabolism of substances and water-salt and change the function of the blood and circulatory system, thereby improving the body’s tolerance and survivability when faced with stressful stimuli [25]. The experiments of Blanchard R J and Zipes D P found that stress triggered a compensatory physiological response in the body, with elevated levels of stress hormones such as ACTH, CRH, E, GC, DA, and other stress hormones [26,27]. In studies related to microwave radiation, some scholars have found that microwaves can cause changes in hormone levels in serum, with elevated acetylcholine (Ach) and epinephrine (E), as well as abnormal cardiac electrophysiological function. According to the literature and model characteristics, a total of six stress hormones were detected in this experiment, among which COR, CRH, ACTH, and GC were the representative hormones of the HPA axis. The results showed that elevation occurred on Day 1 after exposure and did not recover on Day 28, which suggested strong excitation and a slow recovery of the HPA axis, in which the changes in COR and CRH were more pronounced. NE and DA, which are catecholamines, were significantly increased on Days 1, 7, 14, and 28 after exposure, reflecting the strong excitation of the sympathetic-adrenal medulla system, which was presumed to be one of the causes of myocardial structural damage and changes in HRV in the present study. The increase in hormone levels in this study indicated that the cardiac function was damaged, which was an important basis for the presence of physiological stress injury in this model.

Heart rate and the excitability and contractility of the myocardium depend on intrinsic properties of the myocardium, which are modulated by sympathetic and parasympathetic nerves in the intracardiac nodal plexus. During a stress reaction, an appropriate increase in cardiac sympathetic tone leads to an increase in heart rate and myocardial contractility, while parasympathetic nerve suppresses excessive sympathetic excitation. When the stress stimulation is too intense or long, it can bring about excessive activation of the sympathetic nerve and HPA axes, and the secretion of related hormones will also be excessive. Excessive catecholamine hormones and COR can cause a substantial increase in cardiomyocyte autoregulation, an increase in water and sodium retention, myocardial K^+^ reduction, lower ventricular fibrillation threshold, and an increase in the probability of arrhythmia. A large number of studies have shown that stress generated by a certain intensity of stimulation can lead to changes in various cardiac electrophysiological indicators [28]. Furthermore, HRV is significantly changed in stressed patients, thereby increasing the risk of cardiovascular disease [29,30]. Other studies have indicated that HRV could be used to assess the level of stress [31]. A total of ten HRV-related indicators—five time-domain and five frequency-domain related indicators—were analyzed in this study. Among them, heart rate was significantly increased, and mean RR, HRV tri-index, RMSSD, and SDNN were significantly decreased after radiation, suggesting the activity of ANS was off-balance, resulting in sympathetic nerve overexcitation and impaired parasympathetic nerve function. In addition, five frequency-domain related indicators were also evaluated: LF, normalized LF, and LF/HF ratio were significantly increased, indicating enhanced activity of the sympathetic nerve. Additionally, HF and regularized HF were significantly decreased, suggesting diminished parasympathetic activity. Meanwhile, it has been reported that vagal depression was associated with a significant loss of bodily fluids under heat stress [32]. Therefore, we speculated that the changes in HRV noted in this study might be related to fluid loss due to the thermal effect of radiation. The change in HRV occurred on Day 1 after exposure, and all of the time-domain-related indexes recovered by Day 28 after radiation, whereas the three indexes of LF in the frequency-domain analysis had not fully recovered by Day 28, which suggested that the damage caused by electromagnetic radiation has a longer-lasting effect on the ECG signal in the low frequency range. The results of time-domain and frequency-domain analysis verified the excitation of the sympathetic nerve and the inhibition of the vagus nerve, suggesting the impairment of cardiac electrical conduction function, which once again demonstrated the presence of physiological stress injury in this model.

The above experimental results made it clear that physiological stress injury occurred in this model, which was manifested in: the excessive activation of neuro-endocrine, which led to pathological structural damage in the myocardium; abnormal electrophysiological function; and an increase in the level of stress hormones. At the organelle level, mitochondria exhibited structural damage and dysfunction, which led to an imbalance between the oxidative and antioxidant systems of myocardial tissue, resulting in oxidative stress injury. Although mitochondrial function, oxidative stress level, stress-related hormones, and HRV are indicators of physiological stress, these could also indicate the existence of psychological stress injury to a certain extent, though they were not the gold standard for verifying the success of the psychological stress model. Therefore, this study intended to further verify whether the psychological stress model was successfully established through assessing body weight and conducting open field experiments [33]. The weights of rats in Group R were remarkably lower than that those of Group C between Day 1 and Day 7 after radiation. Meanwhile, the results of the open field experiment showed that radiation can cause anxiety and depression in rats. The above results suggested that radiation can cause emotional abnormalities in rats, and that the psychological stress model caused by radiation was established successfully.

After the successful establishment of the stress model, this study intended to detect the changes in important molecules in JNK-related stress pathways in post-radiation cardiac injury and to explore the possible mechanism of injury. Some studies have found that, under heat stress, the expression of JNK in cardiac tissue was significantly increased and activated by phosphorylation, thereby regulating the proteins’ expression of its downstream molecules [34]. The NFATc4 molecule downstream of JNK is related to Ca^2+^ exchange of cardiomyocytes and is abundantly expressed in the myocardium, which has a certain regulatory effect on the electrical conduction function of the heart [35].

HSF1 promotes the expression of Hsp72 under heat stimulation, which could indicate the occurrence of heat stress to a certain extent [36]. This research was designed to explore the changes of JNK pathway-related protein expression by WB, to verify the expression changes again through IF, and to analyze the distribution characteristics of the proteins.

The results of WB and IF showed that the expression of stress proteins was elevated, and the phosphorylation of JNK was increased. The above results made clear that the stress model was successfully built at the molecular level; the increase in HSF1 clarified that the model was a heat-stress model, suggesting that it was related to the thermal effect of electromagnetic radiation; the increase in NFATc4 indicated that the change in HRV might be related to Ca^2+^ transport disorder, which provided a new idea for future regulation research related to damage mechanism.

**Figure 18:**
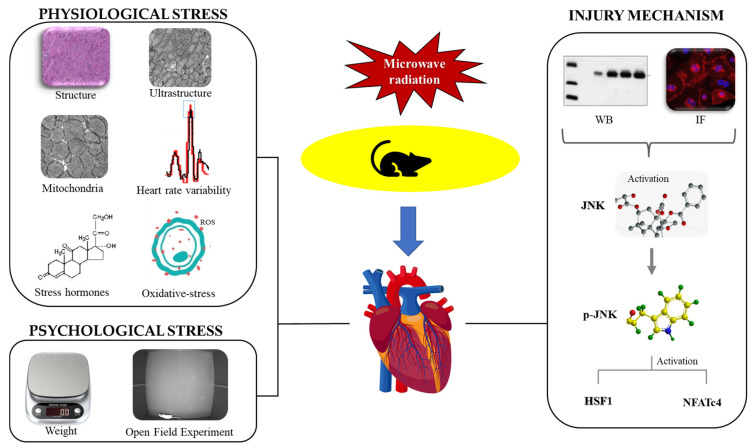
Diagram of research ideology.

To sum up, electromagnetism is used for long-term and high-power applications in communication, medical, and military fields, and the modeling conditions designed in this experiment were designed to simulate the long-term working environment of special electromagnetic occupational groups, expecting to provide new ideas for research on protection. After 35 min of long-term exposure, rats showed an increase in serum hormone, myocardial structural damage, abnormal HRV, myocardial mitochondrial dysfunction, and oxidative-stress damage. The above results demonstrated that physiological stress injury occurred in this model; the psychological stress in this model animal was confirmed by the results of weight and the open field experiment simultaneously; and the mechanism of cardiac injury caused by electromagnetic radiation might be closely related to the activation of JNK pathway. We have plotted the results of this study in Figure 18.

Therefore, this study, from the perspective of stress, investigated the relationship between stress and changes in cardiac structure and function after electromagnetic radiation, and then explored its possible mechanism of injury, which expanded a new orientation for the study of effects in the field, and contributed to providing a new target for protection from radiation. Our study suggested that the cause of cardiac injury from long-term and high-power microwave radiation might be the result of a combination of the thermal and non-thermal effects; therefore, the possibility of cardiac injury from microwaves should be minimized in certain special occupational groups involved in the above environment by shortening the length of a single exposure or extending the interval between exposures as much as possible. In addition, in the study of early warning functional indicators, heart rate variability should be used as a breakthrough with clinical interface for in-depth validation in order to promote application.

## 4. Materials and Methods

### 4.1. Experimental Animals

Fifty-six SPF-grade male Wistar rats (200 ± 20 g) were acquired from Beijing Charles River Center for Experimental Animals. Housing conditions included: ambient temperature (22 ± 1 °C); relative humidity 60%; 3 rats/cage; free feeding and drinking; following circadian rhythm. All procedures were performed under 1% sodium pentobarbital anesthesia (0.5 mL/100 g).

### 4.2. Groups, Microwave Exposure, and Sampling

Fifty-six rats were weighed after 3 d of adaptive feeding, stratified by body weight, and randomly divided into two groups of twenty-eight rats each: the control group (Group C) and the radiation group (Group R).

**Figure 19:**
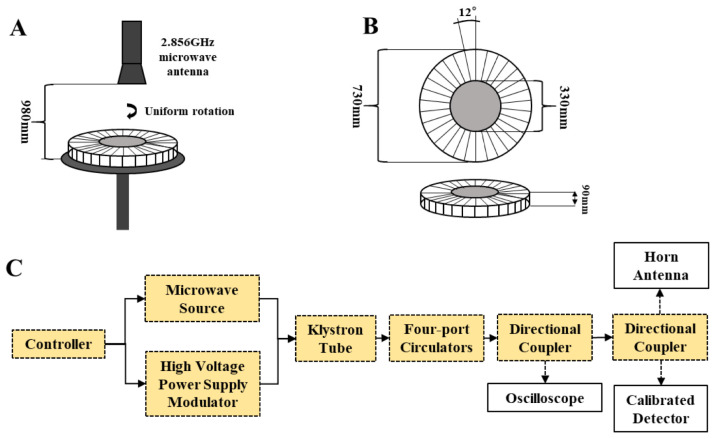
Diagram of the illumination device [15]. (**A**) Radiation table device; (**B**) radiation box; (**C**) microwave generator components and workflow (PD homogeneity of the disc area < 3 dB).

The radiation cassette was made of acrylic in the shape of a disc with thirty individual compartmentalized spaces of the same volume, each with a separate lid. Twenty-eight rats were placed in the twenty-eight compartments of the radiation box with the lid closed, the radiation box was positioned on a radiation table below the radiation source, and the radiation table was rotated to eliminate position effects. The rats in the Group R were radiated with a microwave frequency of 2.856 GHz at 30 mW/cm^2^ for 35 min (peak power density: 200 W/cm^2^, pulse width: 500 ns, repetition rate: 300 Hz), and the rats in Group C were subjected to the same conditions except that they did not receive radiation, as shown in Figure 19 [15].

Five rats/group were anesthetized by intraperitoneal injection of 1% sodium pentobarbital (0.5 mL/100 g) on Days 1, 7, 14, and 28 after radiation. Blood was collected via the inferior vena cava, 3 mL was collected in a coagulation tube, serum was prepared, and hormones were measured. The hearts were soaked and rinsed in pre-cooled saline until there was no residual blood in the heart, and a 1 mm^3^ fresh tissue block from the apical part of the heart was submerged in 2.5% glutaraldehyde and kept for transmission electron microscopy. A soybean-sized fresh tissue block was removed from the apical part of the heart and immersed in pre-cooled RPMI-1640 medium and kept for mitochondrial membrane potential (MMP), and mitochondrial permeability transition pore (mPTP) was prepared. The left half of the tissue was divided in two from the tip of the heart, submerged in 10% formalin fixative, and kept for paraffin sectioning; the right half of the heart tissue was frozen and stored in a −80 °C refrigerator. In addition, on Day 1 after radiation, the hearts of three rats were removed after anesthetization, and the hearts were snap-frozen and kept for frozen sections. The experimental settings used for this study were: temperature at 26 °C, humidity at 24%, pressure at 1023 hPa.

### 4.3. Structure in Myocardial Tissue

Myocardial tissue was soaked in formalin fixative (10%) for 1 week and cut to 5-mm thickness. We followed the following steps: gradient ethanol dehydration, xylene transparency, wax immersion, embedding, sectioning at 5 μm, baking in oven (80 °C) for 2 h, and HE staining. Structural changes were observed under optical microscope (Leica, Wetzler, Germany) and images were acquired.

### 4.4. Ultrastructure in Myocardial Tissue

A 1 mm^3^ fresh block of myocardium at the apex of the heart was fixed in glutaraldehyde fixation (2.5%) for 2 h, and fixed in osmium fixation solution (1%) for 1 h. After gradient ethanol dehydration, acetone transition, Epon812 resin embedding, and semi-thin section positioning, it was made into ultra-thin 70-nm sections. Then, the slices were stained with lead-uranium and observed by transmission electron microscopy (Hitachi, Tokyo, Japan) to find ultrastructural changes and for image acquisition. The total area and cavitation area of mitochondria in the images were calculated by Image J. The mitochondrial cavitation area/total area provided the mitochondrial cavitation ratio, and the mitochondrial cavitation ratio of each group was statistically analyzed.

### 4.5. Mitochondrial Function in Myocardial Tissue

On Days 1, 7, 14, and 28 after radiation, fresh blocks of myocardium soaked in RPMI-1640 medium were cut up with ophthalmic scissors, digested with collagenase (Solarbio, Beijing, China) at 37 °C for 60 min, filtered, centrifuged, resuspended, and centrifuged again. We then added 2 mL of PBS to each tube for the mPTP and MMP experiments, and counted the cells.

#### 4.5.1. Mitochondrial Permeability Transition Pore (mPTP)

We took the appropriate volume of cell suspension and centrifuged for 5 min (2357 r, 26 °C). The supernatant was discarded, and the cells were resuspended by adding the appropriate volume of diluent buffer, Calcein AM staining solution, and fluorescence quenching solution, in order to make a single cell suspension with a cell density of 10^6^/mL. The volume of each sample was 1 mL. The cell sample containing only diluent buffer was used as a negative control for the flow cytometry assay. After 30 min incubation at 37 °C protected from light, the cells were collected by centrifugation for 5 min (2357 r, 26 °C). Each sample was added with 1 mL of diluent buffer, gently resuspended, and cells were collected by centrifugation at 2357 r for 5 min at room temperature, and resuspended with 400 μL of diluent buffer. The above reagents were taken from the Mitochondrial Permeability Transition Pore kit (Beyotime Biotechnology, Beijing, China), and the average fluorescence amount was detected by flow cytometry (Beckman, Shanghai, China).

#### 4.5.2. Mitochondrial Membrane Potential (MMP)

The appropriate volume of cell suspension was taken and centrifuged for 5 min (2357 r, 26 °C) and resuspended using 0.5 mL of RPMI-1640 medium, then 0.5 mL of JC-1 staining working solution was added. The mixture was mixed upside down, incubated for 20 min at 37 °C in the cell incubator, centrifuged for 4 min (1667 r, 4 °C), washed twice using diluent buffer, and finally resuspended with the appropriate volume of diluent buffer. Apoptosis inducer (CCCP:diluent buffer = 1:1000) was added in a positive control tube before incubation; only staining buffer was added to the blank control tube. The above reagents were obtained from the MMP Kit (Beyotime, Shanghai, China), the average fluorescence amount was detected by flow cytometry (Beckman, Shanghai, China), the excitation wavelength was 490 nm, and the emission wavelength was 530 nm.

### 4.6. The Level of Oxidative Stress in Myocardial Tissue

Appropriate amounts of frozen myocardial tissues on Days 1, 7, 14, and 28 after radiation were weighed, added to an appropriate volume of PBS (20 mg/100 uL), homogenized (60 Hz, 60 s, 10 times, 15 s interval) in an automatic homogenizer (Huyi Industrial, Shanghai, China) and centrifuged for 15 min (4 °C, 4000 r. The supernatant was aspirated as protein solution, and a BCA kit (Thermo, Waltham, MA, USA) was used to detect the protein concentration.

#### 4.6.1. Superoxide Dismutase (SOD) Activity

Following the instructions of the kit (Nanjing Jiancheng, Nanjing, China), a 96-well plate was used to set up two control wells, control-blank wells, test wells and test-blank wells, and reagents were added according to Table 1 and mixed well. The 96-well plate was incubated (37 °C) for 20 min and read at 450 nm according to a Microplate Reader (Molecular Devices, Sunnyvale, San Jose, CA, USA).

The inhibition rate of SOD was calculated using the following Formula (1):

(1) $$\mathrm{SOD}\;\mathrm{inhibition}\;\mathrm{rate}\left(\%\right)=\frac{\left(A\;\mathrm{control}-A\;\mathrm{control}\;\mathrm{blank}\right)-\left(A\;\mathrm{test}–A\;\mathrm{test}\;\mathrm{blank}\right)}{A\;\mathrm{control}-A\;\mathrm{control}\;\mathrm{blank}}$$

SOD viability was calculated according to the following Equation (2):

(2) $$\mathrm{SOD}\;\mathrm{viability}\;\left(U⁄\mathrm{mg}\;\mathrm{prot}\right)=\frac{\mathrm{SOD}\;\mathrm{inhibition}\;\mathrm{rate}\;}{\;50\%}\times \frac{\mathrm{Diluted}\;\mathrm{times}\;}{C\;\mathrm{sample}}$$

#### 4.6.2. Malondialdehyde (MDA) Content

We strictly followed the instructions of the kit (Nanjing Jiancheng, Nanjing, China), added reagents according to Table 2, and mixed well. The mouth of the tube was tied with cling film and a small hole was pricked with a needle. The tubes were bathed in water at 95 °C for 40 min, then cooled under running water and centrifuged for 10 min (room temperature, 3500–4000 r). We collected the supernatant, which read at 532 nm according to the Microplate Reader.

The MDA content of tissues was calculated using the following Formula (3):

(3) $$\mathrm{MDA}\;\mathrm{content}\;\left(\mathrm{nmol}/\mathrm{mg}\;\mathrm{prot}\right)=\frac{\left(\mathrm{OD}\;\mathrm{measured}\;-\;\mathrm{OD}\;\mathrm{control}\right)}{\left(\mathrm{OD}\;\mathrm{standard}\;-\;\mathrm{OD}\;\mathrm{blank}\right)}\times \frac{10\;\mathrm{nmol}/\mathrm{mL}\;}{C\;\mathrm{sample}}$$

### 4.7. Stress-Related Hormones in Serum

We took sera from rats on Days 1, 7, 14, and 28 after radiation using radioimmunoassay and operated according to the following steps: (1) We put unlabeled antigen (standard and serum to be tested) into tubes, then added labeled antigen and specific antibody quantitatively in order, mixed well, and performed competitive inhibition reaction under certain conditions; (2) we added an appropriate amount of PR reagent into the tubes, mixed well, and placed it in room temperature conditions. (3) we centrifugated (4 °C, 3500 r) for 20 min, deserted the supernate and gauged the cpm of the precipitate. We then calculated the cortisol (COR), norepinephrine (NE), dopamine (DA), glucocorticoid (GC), corticotropin releasing hormone (CRH), and adrenocorticotropic hormone (ACTH) concentrations in serum according to the cpm.

### 4.8. Heart Rate Variability

On Days 1, 7, 14, and 28 after radiation, the ECGs of rats (5 rats/group) were examined using a polysomnographic recorder and analysis system (BIOPAC, Goleta, CA, USA) with a bioamplifier connected (sensitivity of 2000 Hz). The rats were anesthetized, shaved of their extremities, fixed in supine position, and sterilized. An ECG of standard II leads was recorded continuously for 3 min in the quiet state, and the HRV was analyzed using the time-domain method, including mean heart rate (mean HR), mean RR interval (mean RR), triangular index of HRV (HRV tri-index), root mean square of RR interval difference (RMSSD), and standard deviation of RR interval (SDNN). The frequency-domain analysis indexes included: low-frequency power (LF-ms^2^), high-frequency power (HF-ms^2^), normalized low-frequency power (LF-nu), normalized low-frequency power (HF-nu), and low-to-high frequency power ratio (LF/HF radio).

### 4.9. Weighing before and after Radiation

The rats in the two groups were weighed (Liuyi Instrument Factory, Beijing, China) and their weights were recorded at 14 days, 7 days, and 1 day before radiation and on Days 1, 3, 5, 7, 14, 21, and 28 after radiation to compare the differences in body weight between Group C and Group R at each time point and analyze the effect of radiation on rat’s weight.

### 4.10. The Open Field Experiment and Modification Behavior

**Figure 20:**
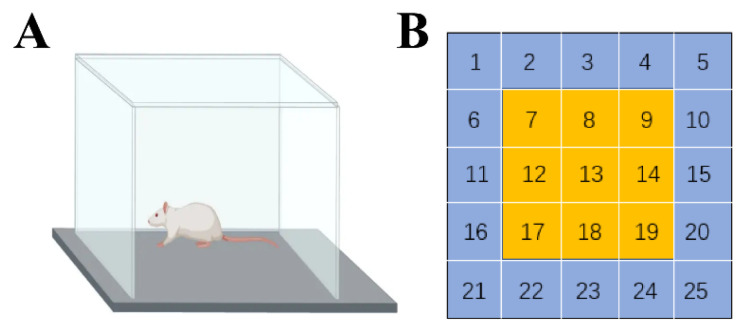
Diagram of the open field experimental setup. (**A**) Diagram of the open field reaction chamber; (**B**) diagram of the bottom partition of the reaction chamber (the bottom partition of the reaction chamber was divided into 25 cells, with the middle 9 cells defined as the central region and the remaining 16 cells as the edge region).

The open field experiments (3 min/rat) were conducted one day before radiation (adaptation), and 6 h, 1 day, 7 days, and 14 days after radiation, and each experiment was preceded by 1 h of adaptation to the experimental room. The experimental setup consisted of a reaction chamber and an automatic data acquisition and processing system (Anymaze, Waunakee, WI, USA). The rat reaction box was 35 cm high, square, and had a 100 cm long bottom side and black interior, with a camera set up directly above it which observed the entire interior of the open field. In the software setup, the bottom partition of the reaction chamber as divided into 25 cells, with the middle 9 cells defined as the central region and the remaining 16 cells as the edge region (Figure 20). The experimental staff and computer equipment were located in another room to minimize the disturbance to the animals, and the background noise of the experiment was controlled at <65 dB. The rats were placed in the center of the bottom surface of the box while the camera and timing were performed. We recorded the total distance moved, total average speed, active time, number of times the rat entered the central region, time spent in the central region, distance moved in the central region, frequency and duration of upright behavior, and frequency and duration of grooming the rats demonstrated in the open field within 3 min. After each rat finished the experiment, we cleaned and wiped the bottom surface of the open field with alcohol to avoid the residual information of the last animal affecting the results of the next test.

### 4.11. Expression and Distribution of Stress Proteins in Myocardial Tissue

#### 4.11.1. WB

The frozen myocardial tissue from Days 1, 7, 14, and 28 after radiation was weighed at 20 mg, added to 200 μL of tissue lysis solution (1:100 with protease inhibitor), homogenized in 60 Hz 10 times (45 s each time, interval 15 s), and centrifugated at 12,000 r in 4 °C for 15 min. The supernatant was collected as total protein solution. The concentration of protein solution was measured using the BCA method, and the loading buffer and protein solution (4:1) were mixed and then denatured at 95 °C for 10 min. After the gel preparation, electrophoresis, membrane transfer, and closure, the anti-JNK (Rabbit, Abcam, ab179461, Cambridge, UK), anti-p-JNK (Rabbit, Abcam, ab219584, Cambridge, UK), anti-NFATc4 (Rabbit, Abcam, ab3447, Cambridge, UK), anti-HSF1(Rabbit, Abcam, ab52757, Cambridge, UK) (diluted to 1:1000 with TBST), and anti-GAPDH (Rabbit, Abcam, ab9485, Cambridge, UK) (diluted to 1:10,000 with TBST) were added to the PVDF membranes and incubated overnight at 4 °C with slow shaking. The next day, the PVDF membranes were washed 3 × 10 min with TBST, added to HRP-labeled secondary antibody IgG (Abclonal, Boston, USA) (diluted to 1:10,000 with TBST), shaken slowly at room temperature for 1 h, and washed with TBST for 3 × 10 min. The targeted proteins were detected by enhanced chemiluminescence (ECL), calculated by Image J.

#### 4.11.2. IF

The frozen tissue was cut into 10-µm slices using a thermostatic frozen slicer (Leika, Wetzler, Germany), and the slices were dried at 26 °C for 15 min. After the fixation, permeabilization, and blocking steps, the slices were dripped with anti-JNK, anti-p-JNK, anti-NFATc4, and anti-HSF1 (diluted in 1:100 by 10% goat serum), incubated overnight at 4 °C, rewarmed for 1 h at 26 °C, and washed for 3 × 5 min in PBS. The fluorescent secondary antibody (Goat, Abcam, Cambridge, UK) (diluted in 1:100 by 10% goat serum) was added to the slices, and all subsequent steps were conducted while protected from light. The slices were incubated at 37 °C for 1 h, washed by PBS for 3 × 5 min, added to anti-quenching blocker containing DAPI, observed under fluorescence microscope, and photographed. Then the photographs were analyzed by Image J.

### 4.12. Experimental Program and Statistical Analysis

**Figure 21:**
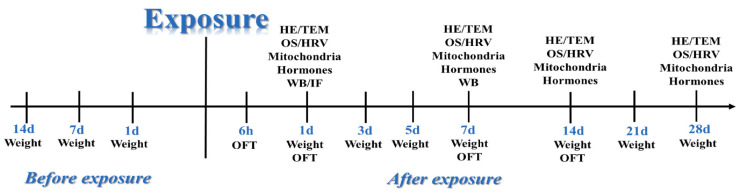
Experimental schedule.

A total of 10 experiments were set up in this study, including: structural and ultrastructural observation, functional testing of mitochondria, detection of oxidative stress’s marker, testing of stress-related hormone in serum, HRV analysis, body weight, the open field experiment, and WB and IF analyses (Figure 21).

Data in the text are presented as mean ± SD ($\bar{X}\;\pm \;S)$ and compared with Group C. Statistical analysis was performed by independent samples *t*-test in SPSS 19.0 version. The acceptable level of significance in the study was *p* < 0.05. Significance signs were classified according to *p*-value: * p < 0.05 and ** *p* < 0.01.

## Figures and Tables

**Table 1:** Reagent composition of each well for SOD vitality detection.

The Liquid Added	Control Wells	Control Blank Wells	Test Wells	Test Blank Wells
Sample to be measured (μL)	-	-	20	20
Double distilled water (μL)	20	20	-	-
Enzyme working solution (μL)	20	-	20	-
Enzyme dilution solution (μL)	-	20	-	20
Substrate application solution (μL)	200	200	200	200

**Table 2:** Composition of reagents for each tube of MDA content detection (mL).

The Liquid Added	Blank Tube	Standard Tube	Measured Tube	Control Tube
Standard (10 nmol/mL)	-	-	20	20
Anhydrous ethanol	20	20	-	-
Test sample	20	-	20	-
Working solution I	-	20	-	20
Working solution II	200	200	200	200

## Data Availability

Not applicable.

## Abbreviations

Ach: Acetylcholine; ACTH: Adrenocorticotropic hormone; AU: Average fluorescence intensity; CRH: Corticotropin releasing hormone; COR: Cortisol; DA: Dopamine; E: Epinephrine; GC: Glucocorticoid; HRV: Heart rate variability; HSF1: Heat shock factor; Hsp72: Heat shock protein 72; HF-ms^2^: High-frequency power; IF: Immunofluorescence; IPSC-CM: Induced Pluripotent Stem Cells Cardiomyocytes; JNK: c-Jun N-terminal kinase; LF-ms^2^: Low-frequency power; LF/HF radio: Low to high frequency power ratio; Mean HR: Mean heart rate; Mean RR: Mean RR interval; MDA: Malondialdehyde; MMP: Mitochondrial membrane potential; mPTP: Mitochondrial permeability transition pore; HF-nu: Normalized high-frequency power; LF-nu: Normalized low-frequency power; NE: Norepinephrine; NFATc4: Nuclear factor of activated T-cell 4; p-JNK: phosphorylated c-Jun N-terminal kinase; RMSSD: Root mean square of RR interval difference; SOD: Superoxide dismutase; SDNN: Standard deviation of RR interval; HRV tri-index: Triangular index of HRV; WB: Western blot.
